# Weight-Loss Cognitive-Behavioural Treatment and Essential Amino Acid Supplementation in a Patient with Spinal Muscular Atrophy and Obesity

**DOI:** 10.1155/2018/4058429

**Published:** 2018-05-17

**Authors:** Marwan El Ghoch, Paola Vittoria Bazzani, Simona Calugi, Riccardo Dalle Grave

**Affiliations:** ^1^Department of Eating and Weight Disorders, Villa Garda Hospital, Garda, Verona, Italy; ^2^Department of Radiology, Villa Garda Hospital, Garda, Verona, Italy

## Abstract

Spinal muscular atrophy is a genetic neuromuscular disease characterised by muscle atrophy, hypotonia, weakness, and progressive paralysis. Usually, these patients display increased fat mass deposition and reductions in fat-free mass and resting energy expenditure—an unfavourable condition that facilitates the development of obesity. However, weight management of these patients remains poorly described. Hence, the aim of this case report was to describe the clinical presentation and weight management of a 31-year-old male patient with spinal muscular atrophy type III, class I obesity, and metabolic syndrome treated for 1 year by means of a personalised multistep cognitive-behavioural treatment for obesity. The treatment produced a weight loss of 7.2 kg (7.1%), which was associated with a marked improvement in both the patient's self-reported general conditions and obesity-related cardiometabolic profile, and no adverse effects in terms of spinal muscular atrophy (i.e., reductions in fat-free mass or resting energy expenditure).

## 1. Introduction

Spinal muscular atrophy (SMA) is a progressive, recessive, inherited neuromuscular disease characterised by muscle atrophy, hypotonia, weakness, and progressive paralysis due to loss of spinal cord motor neurones [1]. It has an estimated incidence of 1 case per 15,000–20,000 live births worldwide and affects both genders at the same rate, although the disease progression is more severe in males [1]. In an effort to predict prognosis, the SMAs have been classified into four categories based on their severity and age of symptom onset, namely, type I, or Werdnig–Hoffmann disease; type II, or juvenile SMA; type III, or Wohlfart–Kugelberg–Welander syndrome; and type IV, whose onset occurs during early adulthood [2].

Patients with SMA usually display higher fat mass (FM) and lower fat-free mass (FFM), as measured by dual X-ray energy absorptiometry (DXA), with respect to the respective reference values for sex and age; indirect calorimetry shows a reduced resting energy expenditure (REE), than that derived from predictive formulas (i.e., Harris and Benedict, and Schofield) [3].

This unfavourable condition unavoidably facilitates the development of overweight and obesity [4], but to date, how to manage the excess weight in these patients remains poorly described and controversial [4]. In particular, the effect of weight loss on SMA is unclear (i.e., pejorative versus beneficial), because during weight loss, in addition to the loss of FM, there is also a loss of FFM that might worsen the SMA muscular status.

In this case report, however, we explore the interaction between SMA type III and obesity in a patient with both conditions who successfully underwent weight management by means of a personalised cognitive-behavioural programme for obesity (CBT-OB) [5]; after one year, the patient displayed improved body composition, metabolic variables, and cardiovascular risk factors, with no adverse effects on his SMA profile.

## 2. Case Presentation

With the patient's consent, we present the case of a 31-year-old male with spinal muscular atrophy type III and class I obesity (body weight 101.4 kg; height 177 cm; BMI 32.0 kg/m^2^) who came to our observation at the Villa Garda Hospital (Italy) outpatient service on 9 March 2016. According to his medical history, at the age of two years, he received a diagnosis of Wohlfart–Kugelberg–Welander syndrome (SMA type III); at the age of 17 years, he underwent an arthrodesis surgical intervention for vertebral collapse and severe scoliosis; at the age of 28 years, he was given a diagnosis of a severe sleep apnoea syndrome, and since then, he has been under bilevel positive airway pressure (Bi-PAP); these ongoing conditions are known to be a consequence of SMA and tend to worsen in the presence of obesity.

Indeed, over the 15 years before coming to our attention, the patient's body weight had progressively increased by about 15 kg to just over 101 kg (his weight at the age of 20 was roughly 85 kg), which indicated the onset of obesity; this was likely due to a forced sedentary lifestyle (the patient was wheelchair-bound) and an alteration in his eating habits (i.e., eating in excess between meals). He reported having undergone several weight-loss attempts (>3) as an outpatient, with unsuccessful results.

The patient, referred to us by his general practitioner, was assessed by means of the Comprehensive Appropriateness Scale for the Care of Obesity in Rehabilitation (CASCO-R) to determine the most appropriate healthcare setting for his weight-loss treatment [6]. The patient had a CASCO-R global score of >25, indicating the appropriateness of residential rehabilitative treatment for obesity, which he voluntarily agreed to. Hence, he was admitted to the Villa Garda Hospital Department of Eating and Weight Disorders on 2 December 2016. At admission, he had a body weight of 101.4 kg (BMI 32.0 kg/m^2^). According to the Italian version of the 17th edition of the Eating Disorder Examination (EDE) interview [7], he had a global score of 0.74, which is <1 standard deviation (SD) above the community mean (i.e., under 1.74); he reported no binge-eating episodes or purging behaviours (i.e., self-induced vomiting and laxative or diuretic misuse) over the preceding three months, which indicated the absence of a binge-eating disorder or other types of eating disorder [7].

Fasting blood samples were obtained on the day of admission (Day 1), and laboratory tests (Table 1) showed raised C-reactive protein (1.46 mg/dl; normal values: <0.1 mg/dl), fibrinogen (433 mg/dl; normal values: 150–400 mg/dl), erythrocyte sedimentation rate (29 mm/h; normal values: 0–20 mm/h), low-density lipoprotein (LDL) cholesterol (121 mg/dl; normal values: <100 mg/dl), insulin (27.8 mcU/ml; normal values: 1.9–23.0 mcU/ml), and HOMA-IR index (5.97; normal values: 0.23–2.5), in addition to reduced high-density lipoprotein (HDL) cholesterol (47 mg/dl; normal values: >60 mg/dl). The clinical presentation was indicative of chronic inflammation in obesity complicated by metabolic syndrome [8].

Resting energy expenditure (REE) (Table 1), measured on the morning of the day after admission (Day 2) using the SensorMedics Vmax Encore 229 system [9], evidenced a measured value of 1,589 kcal/day and a respiratory quotient (RQ) of 0.74. At this time, body composition values (Table 1), measured using a dual-energy X-ray absorptiometry (DXA) scanner (Prodigy Primo Lunar; A223040501) and dedicated software (ENCORE 2009, version 13.31) (General Electric Company, Madison, WI, USA) [10], were FFM (43.85 kg), %FFM (44.8%), FM (52.91 kg), %FM (54.7%), trunk fat percentage (57.0 %), and neck femoral bone mineral density (BMD) (0.655 g/cm^2^).

### 2.1. Personalised Multistep Cognitive-Behavioural Therapy for Obesity

The patient was devised a yearlong personalised CBT-OB programme, beginning with a residential portion lasting 21 days. In addition to daily group CBT sessions, details of which are available elsewhere [5, 11], this programme featured a low-calorie diet of 1,000 kcal/day (55% of which are from carbohydrates, 30% from fat, and 15% from protein). The patient was discharged after three weeks of residential treatment on 21 February 2017, having achieved a weight loss of 3.6 kg (a body weight of 97.8 kg and a BMI of 30.8 kg/m^2^). Immediately after discharge, the patient underwent a one-year standardised outpatient programme based on CBT-OB, during which he was encouraged to continue applying the procedures and strategies learned during his residential stay (e.g., self-monitoring, weekly weighing, meal planning, problem solving, and cognitive restructuring). The outpatient portion of the programme involved 12 individual CBT-OB sessions over the course of one year; the first four of these follow-up sessions were held every 15 days and then the remainder on a monthly basis. These outpatient sessions encompassed both the weight-loss phase (which lasts 6 months) and the subsequent weight-maintenance phase. Throughout the entire treatment (residential and outpatient), the patient took a mixture of essential amino acids (Aminotrofic®) (AFC) 8 g/day (i.e., leucine, lysine, isoleucine, valine, threonine, cystine, histidine, phenylalanine, methionine, tyrosine, and tryptophan with vitamin B6 and vitamin B1) as a dietary supplement.

### 2.2. Treatment Outcomes

The patient completed the CBT-OB programme on 15 December 2017; at this time, his body weight had fallen to 94.2 kg and his BMI to 29.7 kg/m^2^—a weight loss of 7.1%. DXA body composition assessment was repeated and revealed the following values (Table 1): FFM (44.66 kg), %FFM (47.4%), FM (48.46 kg), %FM (52.00%), trunk fat percentage (55.00%), and neck femoral BMD (0.681 g/cm^2^). REE was 1491 kcal/day, RQ was 0.82, and blood values were (Table 1) C-reactive protein (1.13 mg/dl; normal values: <0.1 mg/dl), fibrinogen (413 mg/dl; normal values: 150–400 mg/dl), erythrocyte sedimentation rate (24 mm/h; normal values: 0–20 mm/h), low-density lipoprotein (LDL) cholesterol (106 mg/dl; normal values: <100 mg/dl), insulin (20.9 mcU/ml; normal values: 1.9–23.0 mcU/ml), HOMA-IR index (4.64; normal values: 0.2–32.5), and high-density lipoprotein (HDL) cholesterol (47 mg/dl; normal values: >60 mg/dl).

## 3. Discussion

To our knowledge, this is the first time that a case of weight management in an adult patient with comorbid SMA and obesity has been reported. The case underlines the interaction between the two conditions; SMA is characterised by a loss of muscle mass, which determines a reduction in resting energy expenditure, in turn leading to an increase in body weight and body fat accumulation, especially in the central region. As in our case, the likely outcome of this chain of events is a worsening of obesity and cardiometabolic status [3, 4, 8]. However, in this patient, weight loss via CBT-OB resulted in a significant reduction in total fat mass and improvement in its distribution, with a consequent improvement in inflammatory status and obesity-related metabolic syndrome components. It is important to note that this intervention was not at all harmful in terms of deterioration in fat-free mass, bone mineral density (no changes in BMD), or resting energy expenditure—potential adverse effects of weight loss that may be expected in the case of spinal muscular atrophy.

It is possible that dietary supplementation via a mixture of essential amino acids (AFC) may have played a positive preventive role that counterbalanced the loss of muscular mass, through two potential mechanisms: (i) the activation of protein syntheses, via expression of peroxisome proliferator-activated receptor gamma coactivator 1-alpha (PGC-1*α*) and promoting mitochondrial biogenesis [12], and (ii) the regulation of neuromuscular autophagic signaling via changes in serum essential/nonessential amino acid ratio [13]. However, since these findings are derived from animal and *in vitro* models, further studies in humans are required to test this hypothesis. That being said, it is known that a high intake of leucine reduces catabolism and promotes protein synthesis, especially during weight loss [11]. Moreover, in the context of malnutrition and anorexia in chronic renal patients, branched-chain amino acids have been favourably associated with a significant antidepressant effect by interfering with cerebral serotonin activity and by inhibiting the hyperpressure of the proteolytic muscle metabolic routes [14]. In addition, in an early trial carried out on 25 patients suffering from rheumatic cachexia, the same AFC mixture used in our patient was administered for 12 weeks and induced an increase in fat-free mass and total proteins, as well as an improvement in the patients' overall physical condition [15]. Furthermore, another recent trial that involved 41 patients with muscle wasting demonstrated that AFC was capable of increasing the fat-free mass (assessed using DEXA) and significantly reducing some insulin resistance markers, such as TNF alpha [16].

Our case report therefore has clinical implications for patients with SMA. In particular, regarding the success of our weight-management programme based on personalised multistep CBT-OB (with the first phase of the treatment delivered in a residential setting), which helped the patient to improve adherence to a low-calorie diet, this is especially noteworthy as previous weight-loss attempts based on standard dietary prescription had failed. In addition to being safe, the CBT-OB delivered produced a healthy weight loss and weight maintenance, without reduction in the patient's muscular mass—considered an extremely demanding task in this population—over one year of follow-up, associated with improved general conditions and cardiometabolic status.

This case report has several strengths. In particular, the resting energy expenditure and body composition were measured using indirect calorimetry and DXA, which are known to exhibit high levels of accuracy [9, 10]. Moreover, it is the first to describe successful management of a patient with obesity and SMA exclusively in a specialised unit for obesity and proposes a safe nutritional approach that may flank any other therapeutic procedures in this population. However, it is necessary to underline also that the peculiar care and attention provided to the follow-up of our patient was certainly pivotal in both compliance to the treatment and the good outcome obtained. That being said, the data gathered relate to only one patient, and further data derived from a greater number of patients over a longer period of time will be necessary to confirm the efficacy of our approach in SMA. Nevertheless, our results do indicate that a one-year personalised CBT-OB programme with associated AFC supplementation may be a feasible approach to managing patients with comorbid SMA and obesity, producing a healthy weight loss and maintenance and determining improvements in the obesity-related cardiometabolic profile, with no adverse effects on the SMA status (i.e., no reduction in fat-free mass or resting energy expenditure).

## Figures and Tables

**Table 1:** Body composition, resting energy expenditure variables, and laboratory results on the morning after overnight fasting.

Variable	1st assessment during residential treatment	2nd assessment at the end of outpatient treatment (1-year follow-up)
Fat-free mass percentage (%)	44.80	47.40
Fat-free mass (kg)	43.85	44.66
Fat mass percentage (%)	54.7	52.0
Fat mass (kg)	52.91	48.47
Trunk fat percentage (%)	57.00	55.00
Neck femoral BMD (g/cm^2^)	0.655	0.681
Measured resting energy expenditure (mREE) (kcal/d)	1589	1491
Respiratory quotient (RQ)	0.74	0.82
Creatinine (mg/dl)	0.10	0.10
Glucose (mg/dl)	87	90
Insulin (*μ*U/ml)	27.8	20
HOMA index	5.97	4.64
Total cholesterol (mg/dl)	184	169
HDL cholesterol (mg/dl)	47	47
LDL cholesterol (mg/dl)	121	106
C-reactive protein (mg/dl)	1.46	1.13
Fibrinogen (mg/dl)	433	413
Erythrocyte sedimentation rate (mm/h)	29	24
